# Carbon nanotube-cellulose ink for rapid solvent identification

**DOI:** 10.3762/bjnano.14.44

**Published:** 2023-04-26

**Authors:** Tiago Amarante, Thiago H R Cunha, Claudio Laudares, Ana P M Barboza, Ana Carolina dos Santos, Cíntia L Pereira, Vinicius Ornelas, Bernardo R A Neves, André S Ferlauto, Rodrigo G Lacerda

**Affiliations:** 1 Departamento de Física, Universidade Federal de Minas Gerais, Belo Horizonte - CEP 31270-901, Brazil; 2 CTNano-UFMG - Centro de Nanotecnologia em Nanomateriais e Grafeno, Universidade Federal de Minas Gerais, Belo Horizonte - CEP 31270-901, Brazil; 3 Departamento de Física, Universidade Federal de Ouro Preto, Ouro Preto - CEP 35400-000, Brazil; 4 Centro de Engenharia, Modelagem e Ciências Sociais Aplicadas, Universidade Federal do ABC, Santo André - CEP 09210-580, Brazil

**Keywords:** carbon nanotube, electronic tongue, fibrillated cellulose, liquid sensor

## Abstract

In this work, a conductive ink based on microfibrillated cellulose (MFC) and multiwalled carbon nanotubes (MWCNTs) was used to produce transducers for rapid liquid identification. The transducers are simple resistive devices that can be easily fabricated by scalable printing techniques. We monitored the electrical response due to the interaction between a given liquid with the carbon nanotube–cellulose film over time. Using principal component analysis of the electrical response, we were able to extract robust data to differentiate between the liquids. We show that the proposed liquid sensor can classify different liquids, including organic solvents (acetone, chloroform, and different alcohols) and is also able to differentiate low concentrations of glycerin in water (10–100 ppm). We have also investigated the influence of two important properties of the liquids, namely dielectric constant and vapor pressure, on the transduction of the MFC-MWCNT sensors. These results were corroborated by independent heat flow measurements (thermogravimetric analysis). The proposed MFC-MWCNT sensor platform may help paving the way to rapid, inexpensive, and robust liquid analysis and identification.

## Introduction

The development of a new generation of smart sensors that allow for the monitoring of industrial processes in real time and for wearable and flexible devices are paradigms of the current “Industry 4.0”. One can envision applications such as multicomponent liquid and gas sensors, wearables for healthcare, paper-based sensors, and electronic solutions for smart city applications [1–5]. Another area of increasing demand is the rapid test, identification, and monitoring of liquid samples in various fields such as fuel adulteration, water quality, solvents, and beverages [6–9]. Usually, the testing of liquids requires conventional analytical techniques such as absorption/emission spectroscopy (AAS/AES), X-ray fluorescence spectroscopy, and inductively coupled plasma mass spectrometry (ICP-MS). These techniques are complex, expensive, and require experts to carry them out. Also, they often require several pretreatment steps with high-cost materials [10–11]. Electronic tongues are a category of liquid sensors that could solve these issues. These devices comprise an array of non-specific sensors from which, with an appropriate method of multiple data processing, the desired information can be extracted, constituting one of the promising candidates for developing smart sensor technologies [12–18]. Additionally, the Internet of Things (IoT) also requires devices to be integrated into a variety of systems and different surfaces of our daily life, which demands the low-cost, reliable, and large-scale production of sensors [12–13,19]. Yet, the lack of such reproducible large-scale production of liquid sensors, besides the constant need for sensor recalibration, has hindered broader commercialization of such devices [13,20].

A wide variety of materials have been explored for liquid sensing. For instance, electrically conductive polymer composites (CPCs), which are generally composed of lightweight materials comprising a conductive ingredient (e.g., carbon nanotubes (CNTs), graphene, graphene oxide, and metal particles) embedded in a polymer matrix, have been extensively studied as liquid sensors [14–17,21–22]. The main idea is to combine the responsive electrical properties of carbon nanostructured materials with the polymer’s distinguished mechanical properties. These composites are usually non-selective and can react to various ambient stimuli [20,22–29]. Among polymers, cellulose is the most abundant natural organic polymer on earth. It has resurfaced recently as a smart material because of its excellent thermal-mechanical properties, biocompatibility, biodegradability, and flexibility [22–23,30–31]. Composites based on carbon nanotubes or graphene and cellulose have been reported for, among other things, humidity and vapor sensing, as electromagnetic shielding, and as thermoelectric material [32–38]. Also, Qi et al. reported a liquid-water sensor based on carbon nanotube–cellulose composite films, and, more recently, Goodman et al. reported the scalable manufacturing of nanocomposites for liquid sensing [39–40]. Besides, graphene films deposited on cellulose paper and a graphene/cellulose composite were also reported as a solvent sensor material [30,33]. However, most of these works rely on cellulose as a paper substrate or as a thick composite film that cannot be readily employed for large-scale production.

Ink printing technology is one of the most promising approaches to fulfill all the demands and to mitigate the issues described above. This naturally leads to the challenge of developing new smart-ink-based materials for several applications [1–5,12–13,41–43]. Carbon nanotubes and other 1D/2D materials have been employed as ink components with great potential for a broad range of applications, for example, in flexible electronics, photoconductors, transparent conductors, and gas sensors [44–47]. Carbon nanotube ink films have been reported as field-effect transistors, transparent conductors, gas sensors, supercapacitors, and pH sensors [41–42,47–55]. Different approaches to ink printing methods have been explored, such as aerosol jet, inkjet, syringe, roll-to-roll printing, and stamp methods [1,41,50].

In this work, we report a sensor based on a carbon nanotube–cellulose ink that proves to be highly sensitive to various solvents and water with different impurity levels and can detect glycerin in water down to the 10–100 ppm range. We provide insights into the liquid detection mechanism, combining the well-known swelling mechanism of polymer composites with physicochemical characteristics such as dielectric constant, specific heat, and vapor pressure [25,56–57]. Our ink-based devices could extract those characteristics even from unknown samples and mixtures. Finally, test analysis using principal component analysis (PCA) was performed in different devices and on flexible and rigid substrates, providing a step forward towards scale-up and commercialization of the technology.

## Experimental

### Materials and apparatus

Microfibrillated cellulose (MFC) with a nominal fiber width of 50 nm and several hundred micrometers of length was purchased from Maine University (3.0 wt % aqueous gel) [58]. Further characterizations and information about the MFC can be found in [58]. Functionalized multiwalled carbon nanotubes (MWCNTs) with hydroxy and carboxyl groups (–OH and –COOH), outer diameter between 20 and 50 nm and an average length of 5 μm were produced at CTNano/UFMG [59–61]. Morphological analysis was carried out by scanning electron microscopy (SEM) in a Quanta 200 FEG, using secondary electrons between 2 and 10 kV. Atomic force microscopy (AFM) was carried out on a Bruker MultiMode8 SPM using the intermittent contact mode. AC160TS silicon cantilevers from Olympus with a typical spring constant of *k* ≈ 46 N/m, a nominal radius of curvature of *r* ≈ 7 nm, and a resonant frequency of ω_0_ ≈ 300 kHz were employed. Heat flow and weight changes of selected solvents were determined by thermogravimetric analysis (TGA) using a PerkinElmer STA 8000 device. Electrical measurements were performed using a lock-in amplifier (SR830 DSP Stanford Research Systems), a pre-amplifier (model 1211 DL instruments), and a multimeter (model 2000 Keithley), which were controlled by a computer.

### Conductive ink and conductive polymer composite

MWCNTs were mixed with DI water (1% w/v) and sonicated in an ultrasonic bath for 2 h. The obtained suspension was centrifuged for 5 min at 2500 rpm, and the supernatant (0.6% w/v) was reserved. MFC was dispersed in DI water (0.5% w/v) using a Silverson homogenizer (10,000 rpm) for 10 min and then filtered through a 50 μm sieve, resulting in 0.3% w/v MFC dispersion. Finally, the two suspensions were mixed in 1:1 v/v proportion and homogenized in a Silverson homogenizer to produce the final composite conductive ink, which will be called (MFC/MWCNT). To estimate the solid content of the suspension, a weighed sample of the substance mixture was taken and heated up to 60 °C for a few hours in order to evaporate the residual moisture. The remaining dry residue was weighed and proportioned, yielding the dry substance content of the mixture. See Supporting Information File 1 for details.

### Electronic tongue device: transducer and data acquisition

**Figure 1:**
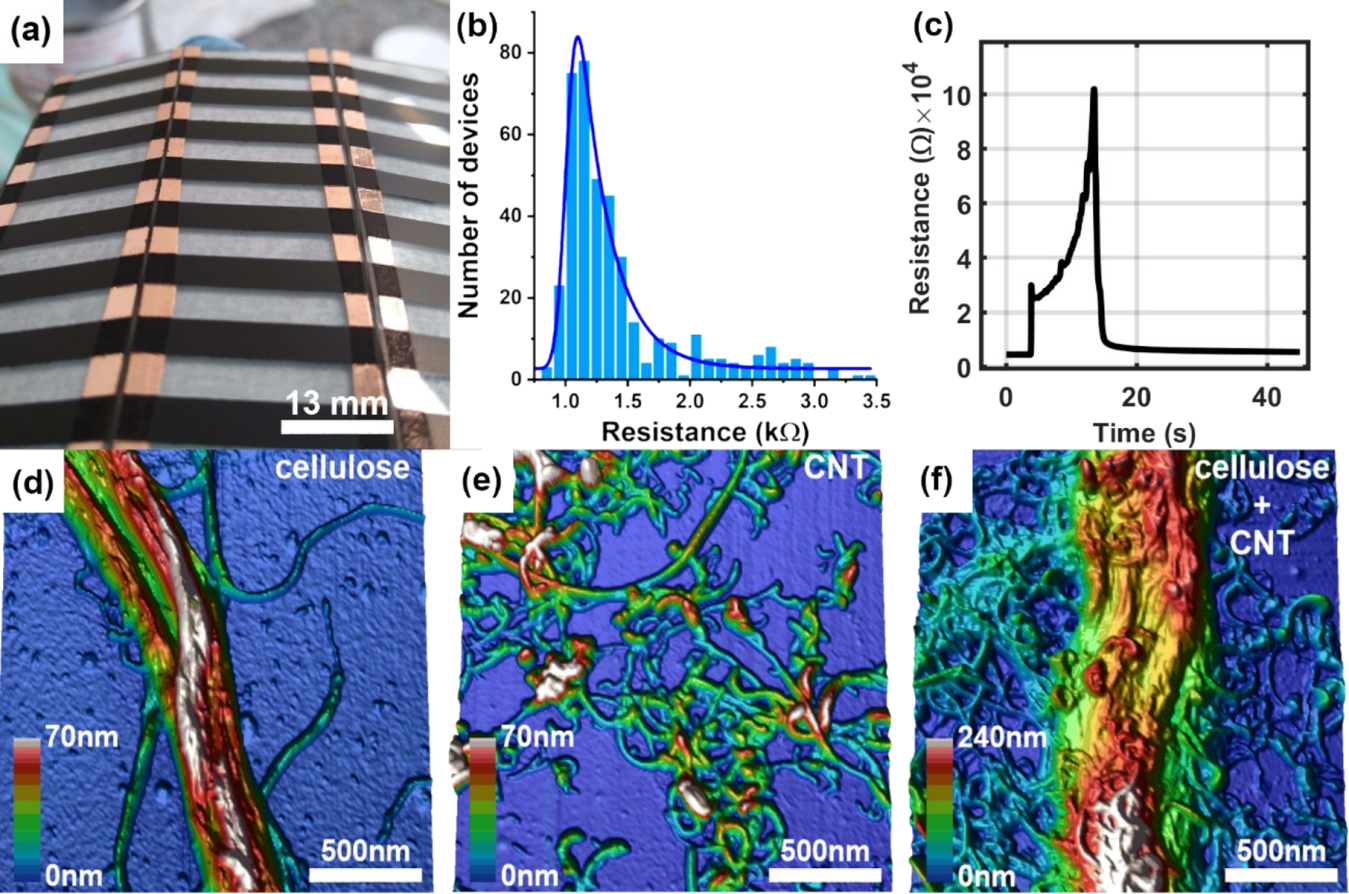
(a) Array of transducers; (b) histogram of sensor resistance distribution; (c) transducer resistance change as a function of time during liquid sensing; AFM images of (d) microfibrillated cellulose (MFC), (e) MWCNTs, and (f) MFC entangled with MWCNTs.

Transducer arrays were produced by spraying the MFC/MWCNT ink onto glass substrates using an airbrush and masking tape as a stencil, as shown in Figure 1a (each black rectangle is an individual sensor). The substrates were kept at 110 °C to speed up water evaporation during painting, preventing the formation of circular drying stains or “coffee rings” patterns and providing thickness control.

After painting, electrical contacts were applied at the ends of each device with conductive silver paint. Thus, series of devices can be prepared that can be varied by changing the number of painted layers. Figure 1b shows a histogram of the initial resistance distribution of the sensors averaged over around 400 devices, demonstrating the system’s robustness for large-scale production. The resistance distribution was fitted using an exponentially modified Gaussian (EMG) and the calculated mean resistance (with standard deviation) is 1.26 ± 0.07 kΩ. A geometry optimization was also performed to find out the maximum gain response as a function of width, length, and number of painted layers (see Supporting Information File 1 for details).

To better understand how MWCNTs and MFC are distributed within the ink, AFM measurements were performed on the isolated materials (MFC and MWCNTs) and on the MFC/MWCNT composite (see Figure 1d–f). Pure MFC fibers form bundles (ca. 250 nm thick), and the functionalized tubes also form small bundles. Interestingly, Figure 1f shows that the carbon nanotubes tend to twine around the MFC fiber when mixed. Thus, one can visualize that the composite ink is composed of an insulating matrix of MFC fibers intertwined by a conductive CNT network.

To analyze the data, principal components analysis (PCA) was performed. PCA is a multivariate technique that transforms several variables correlated with each other in a new set of orthogonal variables (the principal components) to extract and condense the variance information of the whole set in just two or three components, showing the similarities and differences between the classes in the set [62].

## Results and Discussion

### Liquid analysis

#### Detection of glycerin in water

The liquid sensing measurements were performed by applying a fixed voltage (5 V) on the device while measuring the current (*I*) as a function of the time. 6.5 µL of the tested liquid was dripped onto the transducers, while the current was monitored until complete evaporation of the liquid. The device temperature was kept just below the boiling point of the liquid under evaluation. Afterward, the current was used to calculate the resistance, *R*_0_ (see Figure 1c), and the sensitivity gain (*S*), defined as *S* = ((*R* – *R*_0_)/*R*_0_)·100, where *R*_0_ is the initial sensor resistance and *R* is the measured resistance. Features of the experimental curves such as area, maximum peak value, and peak width, were used as input parameters and are described in detail in Supporting Information File 1. We analyzed two different groups of liquids, namely mixtures of water with low concentrations of glycerin (10 and 100 ppm) and a number of organic solvents (DI water, isopropyl alcohol, toluene, chloroform, and ethyl alcohol).

**Figure 2:**
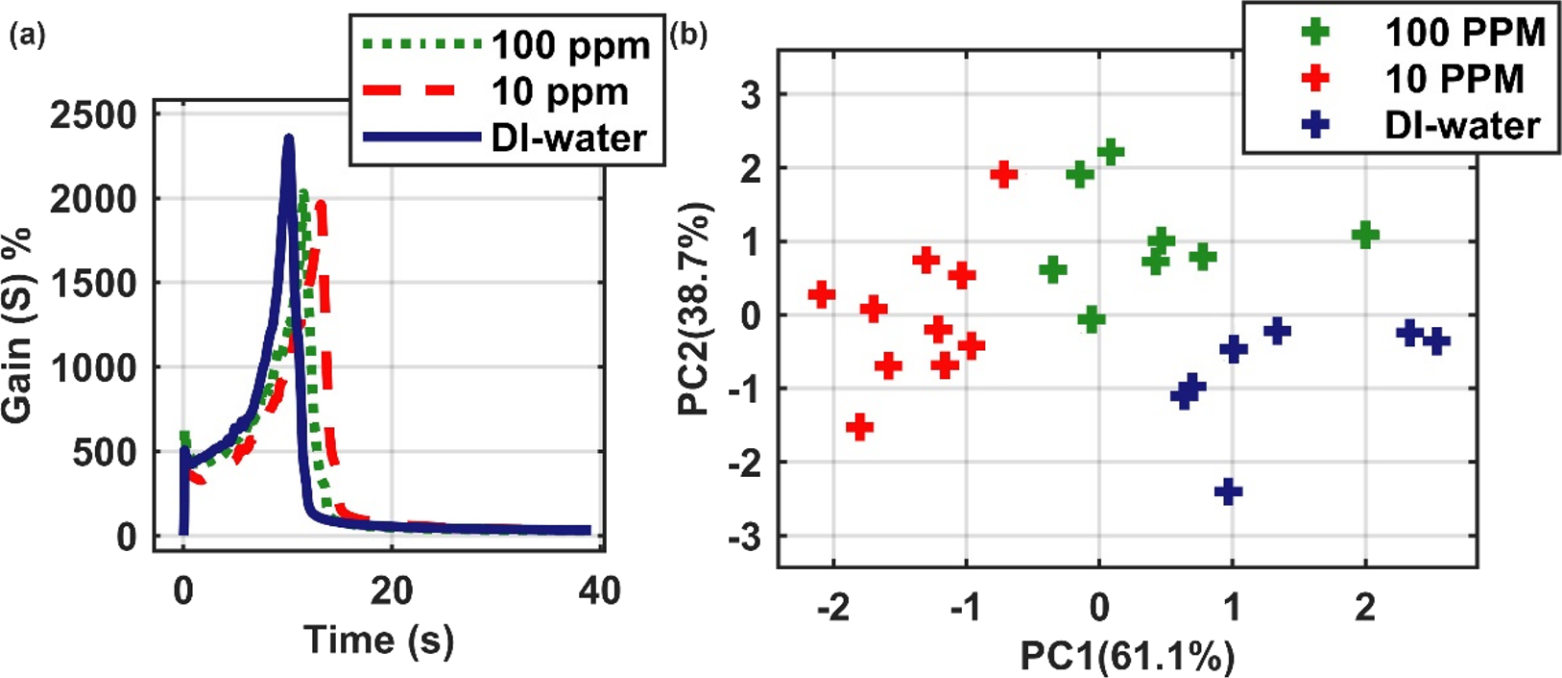
(a) Gain curves obtained from different glycerin/water mixtures; each point at the PCA diagram (Figure 2b) was calculated from a single measurement. (b) PCA analysis of DI water, 10 ppm and 100 ppm glycerin/water mixture.

To probe the limit of detection of the MFC/MWCNT composite, we performed measurements of glycerin in water at parts-per-million concentrations with the sensor temperature set to 95 °C. Since glycerin does not evaporate at 95 °C, it leaves residues in the sensor matrix, preventing the same sensor device to be used in successive measurements. Thus, in this case a single (drop) measurement was performed for each individual sensor. The parameters analyzed by PCA were “max”, “t_max”, “slope”, and “ratio_maxmin” (defined in Supporting Information File 1 as the input features). Figure 2a and Figure 2b depict, respectively, the sensitivity gain *S* (for pure water, and 10 and 100 ppm of glycerin in water) and the PCA analysis for these systems. The sensor was able to distinguish the three cases, demonstrating both robustness and sensitiveness of the MFC/MWCNT composite as a low-concentration oil sensor.

### Organic solvent recognition

**Figure 3:**
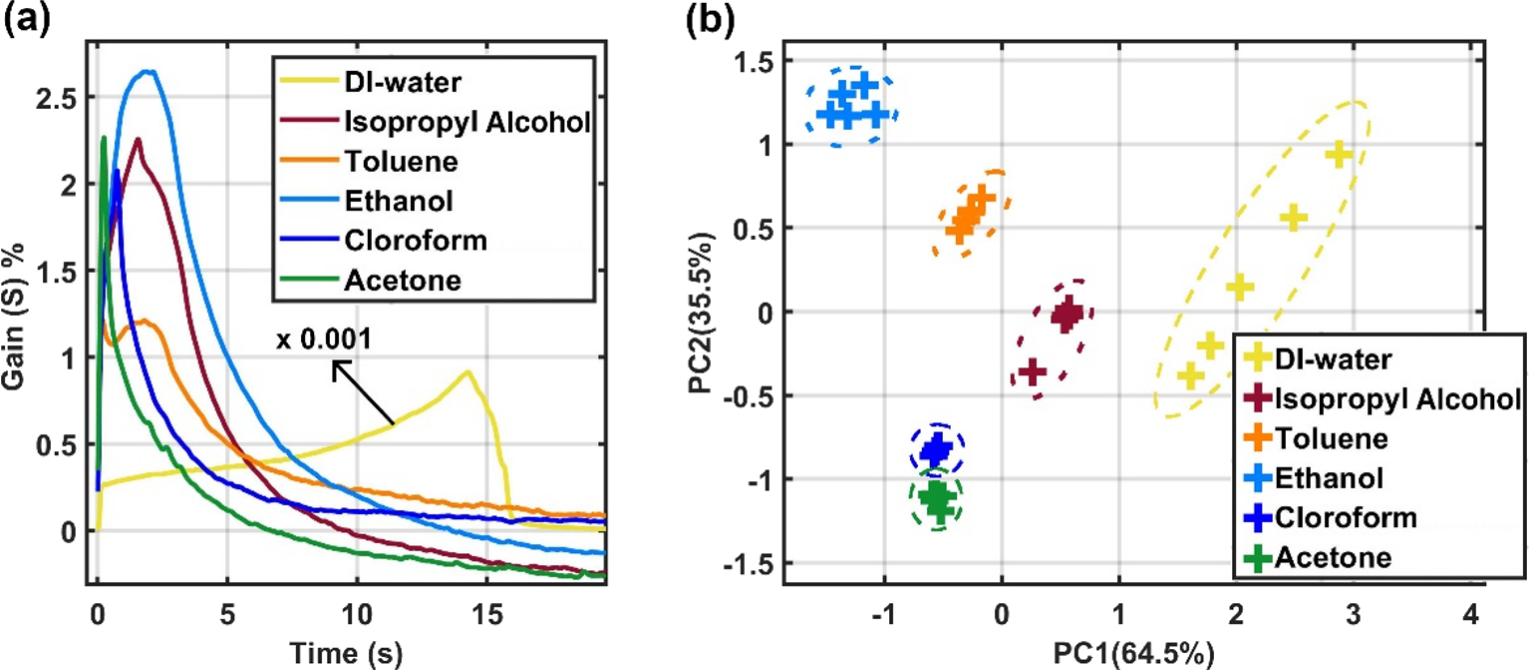
(a) Gain curves obtained from different organic solvents. (b) PCA analysis of organic solvents showing excellent distinction between the sample categories. In the case of DI water, a scale factor of (×0.001) was applied to the curve of Figure 3a to help visualization.

The MFC/MWCNT transducer was also evaluated for the recognition of organic solvents. The electrical responses to DI water, isopropyl alcohol, toluene, ethyl alcohol, chloroform, and acetone are shown in Figure 3a. We set the device temperature to 55 °C to prevent instantaneous evaporation of the more volatile solvents. All solvent measurements were performed on the same MFC/MWCNT transducer. Figure 3a and Figure 3b show the gain as a function of the time and the PCA analysis for all solvents, respectively. Again, all solvents were easily discriminated via PCA analysis. In this case, the parameters used for PCA analysis were area and FWHM with λ = 0.50 (see Supporting Information File 1 for more information). Also, to compare our results with a simple conductive response to the pure liquids, we performed control experiments on a substrate without applying the ink. Our sensor film proved to be two orders of magnitude more sensitive than the bare substrates with only electrical contacts. Further details can be found in Supporting Information File 1.

### Electronic tongue mechanism

**Figure 4:**
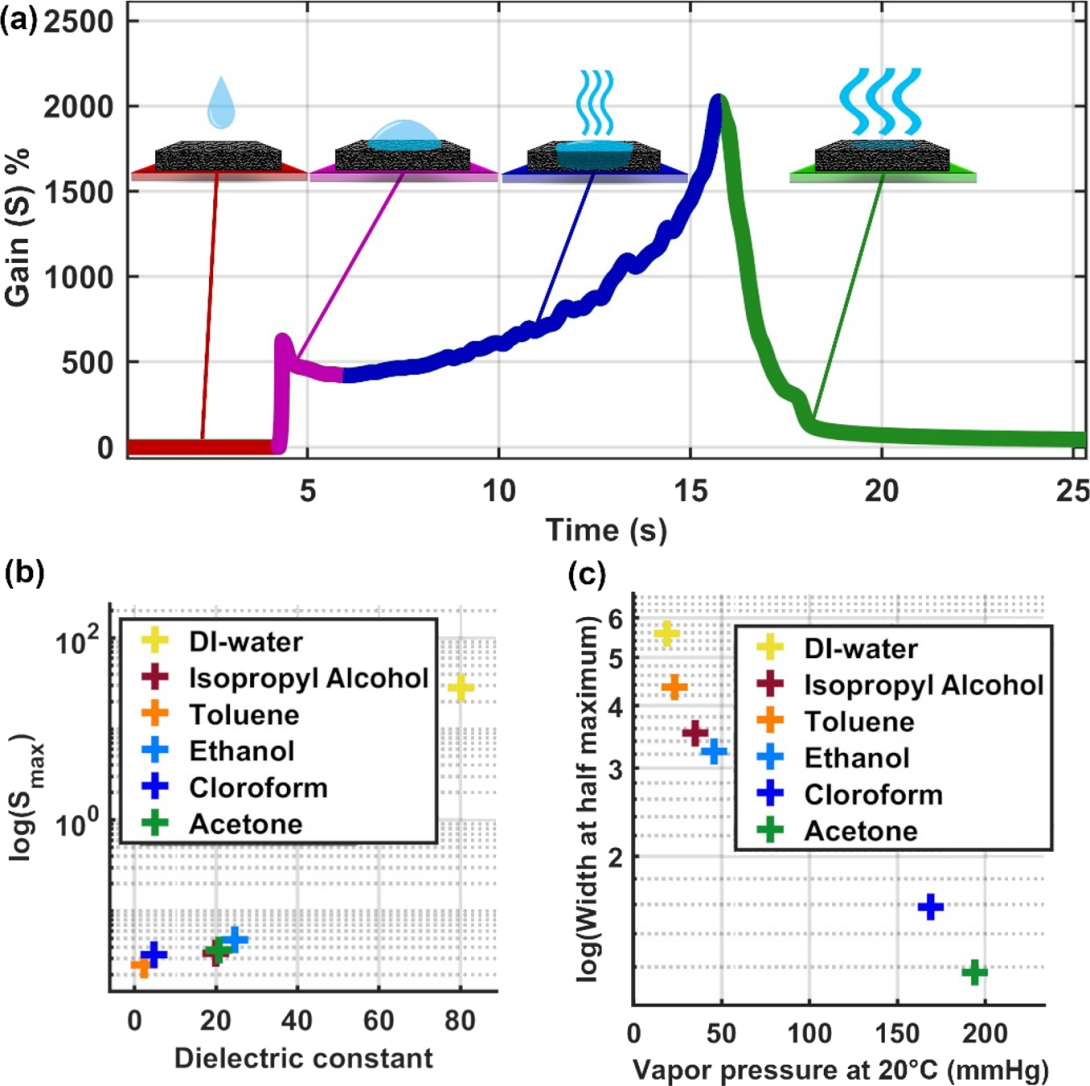
(a) Sketch depicting the step-by-step process of sensing. (b) Correlation between the dielectric constant of the tested solvents and the maximum gain obtained from the Gain curves shown in Figure 3. (c) Correlation between the vapor pressure of the tested solvents and the width at half maximum (FWHM) obtained from the Gain curves shown in Figure 3.

Figure 4a shows the behavior of a liquid as a function of the time as it gets in contact with the transducer. Initially, the composite is dry, at a constant temperature, and traversed by a constant current (red part). As a drop gets in touch with the composite, the electrical current rapidly decreases, and the system starts losing heat as the liquid gets absorbed in the entangled composite matrix. This effect reduces the percolation between the conductive MWCNT clusters, generating an increase in gain (resistance) and a decrease in current (pink part). The absorbed liquid makes the material swell while it simultaneously absorbs heat and evaporates (blue part). At a certain point, the evaporation leads to the drying of the composite, reversing the swelling process. This leads to a decrease in gain until it reaches a point close to its initial value (green part).

In order to understand the nature of the interaction between the transducer and the liquids, we correlated the main variables used for PCA analysis (maximum gain and full width at half-maximum (FWHM) as described in Supporting Information File 1) with physicochemical properties of the organic solvents (vapor pressure and dielectric constant) [63–64]. Guided by previous studies that show the electrical response is due to the swelling of CPCs [25,56–57], we found that the maximum value in the Gain curve (max) is proportional to the dielectric constant of the solvent as shown in Figure 4b. This result suggests that, when the liquid soaks the composite, it swells the material, creating a liquid dielectric barrier between the conductive clusters. Thus, it changes the tunneling process proportionally to the dielectric constant of the liquid. Hence, our results demonstrate that the dielectric constant plays an essential role in the sensing mechanism with a clear correlation with the maximum gain of the sensor.

**Figure 5:**
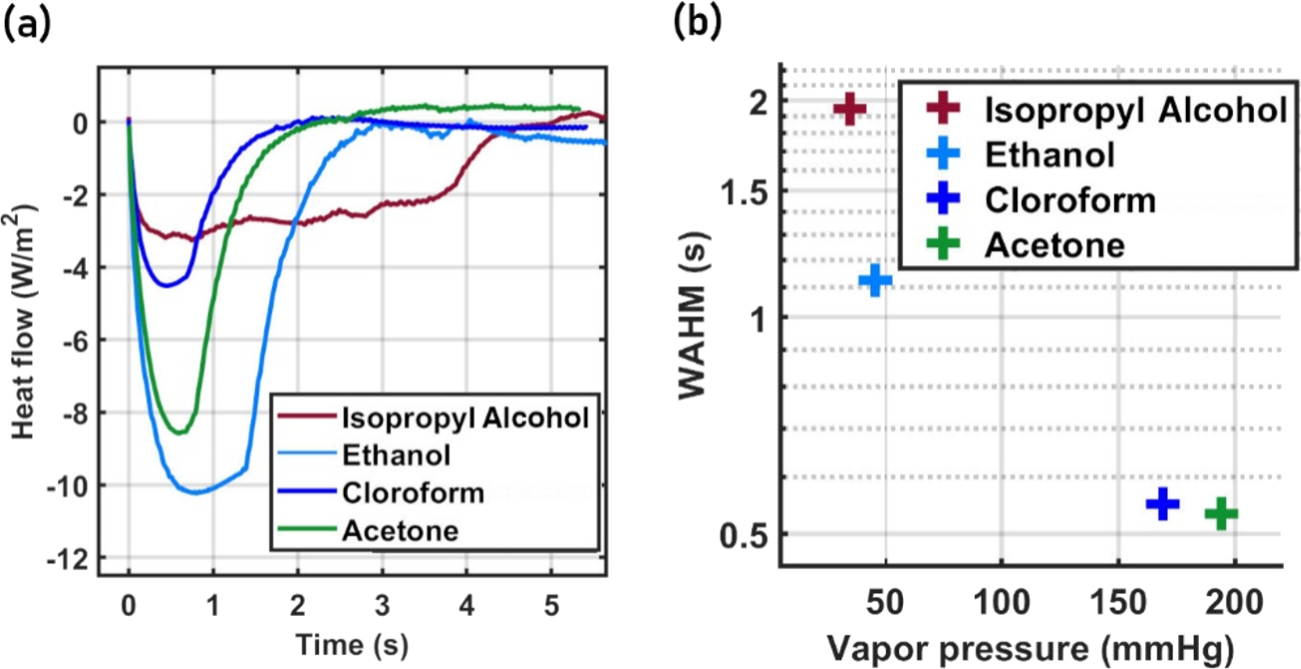
(a) Heat flow behavior of different solvents. (b) Correlation between the width at half maximum (FWHM) and the vapor pressure of the tested solvents.

Also, the width of the curves seems to be strongly influenced by the thermal properties of the liquids. As shown in Figure 4c, the vapor pressure of the solvents controls the time the liquid will stay within the sensor before it evaporates. This makes it a key factor regarding the swelling process of the CPC matrix and the electron tunneling process. To investigate this hypothesis, we designed an experiment to mimic the thermal effects produced by the impinging liquid drops over the heated surface of the transducer. The temperature change and the heat flow produced by the liquid as it gets in contact with the heated surface were estimated by dripping a liquid (of about 6.5 µL) into an empty crucible kept at 55 °C inside a thermogravimetric analyzer. The system was monitored until the complete evaporation of the liquid, resulting in the curves shown in Figure 5a.

The thermal behavior of acetone, chloroform, ethanol, and isopropyl alcohol exhibits significant similarities with the electrical curves presented in Figure 3a, especially regarding the width of the peaks. The interaction of the liquid with the hot crucible is analogous to the interaction of the liquid with the transducer from the thermal point of view. In both cases, the contact of the liquid with the hot surface causes a temperature drop due to the heat transfer from the surface to the fluid. However, as the liquid reaches thermal equilibrium with the surface (minimum point), the heat flow changes direction and the temperature of the system increases, favoring the evaporation of the fluid. Thus, in Figure 5b, we observe a strong correlation between the width of the heat flow curves and the vapor pressure of the liquids. We observed the same correlation for the device curves presented in Figure 4c, evidencing that the thermal proprieties of the liquids are a critical component of the sensors’ electrical response.

## Conclusion

In this work, a liquid sensor was developed based on an easily up-scalable MFC/MWCNT composite manufactured by printing techniques. AFM measurements show that the composite coating consists of an insulating MFC matrix decorated with a conductive CNT network. The sensor response to different liquids and solvents is fast (40 s) and highly reproducible. The glycerin/water experiment shows sensitivity to detect oil compounds down to the parts-per-million range. Also, we demonstrate the important role of dielectric constant and vapor pressure in the transduction mechanism of the MFC/MWCNTs composite. We believe that our sensor can overcome the scale-up and reproducibility limitations of other liquid sensor devices and has great potential to be applied in various industrial fields for liquid monitoring.

## Supporting Information

File 1 Additional experimental data.
