# Supplementary material for: Carbon nanotube-cellulose ink for rapid solvent identification
Source: Beilstein J Nanotechnol. 2023 Apr 26;14:535–43. doi: 10.3762/bjnano.14.44 (PMC10155625; doi:10.3762/bjnano.14.44)
Supplement: File 1 — Additional experimental data. [file Beilstein_J_Nanotechnol-14-535-s001.pdf]

BEILSTEIN JOURNAL OF NANOTECHNOLOGY

Supporting Information

for

Carbon nanotube-cellulose ink for rapid solvent identification

Tiago Amarante, Thiago H. R. Cunha, Claudio Laudares, Ana P. M. Barboza, Ana Carolina dos Santos, Cíntia L. Pereira, Vinicius Ornelas, Bernardo R. A. Neves, André S. Ferlauto and Rodrigo G. Lacerda

Beilstein J. Nanotechnol. **2023,** *14,* 535–543. doi:10.3762/bjnano.14.44

Additional experimental data

License and Terms: This is a supporting information file under the terms of the Creative Commons Attribution License (https://creativecommons.org/licenses/by/4.0). Please note that the reuse, redistribution and reproduction in particular requires that the author(s) and source are credited and that individual graphics may be subject to special legal provisions.

The license is subject to the *Beilstein Journal of Nanotechnology* terms and conditions: (https://www.beilstein-journals.org/bjnano/terms)

Supporting Information – section 1

The water-based carbon nanotube (CNT) ink was produced by directly dispersing functionalized CNTs in the water. The CNTs were produced by chemical vapor deposition (CVD) [1], and the functionalization was carried out via a modified oxidation process, as reported elsewhere [2]. The CNTs were poured in water at a concentration of 1% (w/v) and dispersed under sonication for 3 h. The resultant suspension was centrifuged until a homogenous ink was obtained, with concentrations between 0.4% and 0.6% (w/v). We present some characterizations of the CNTs in Figure S1.

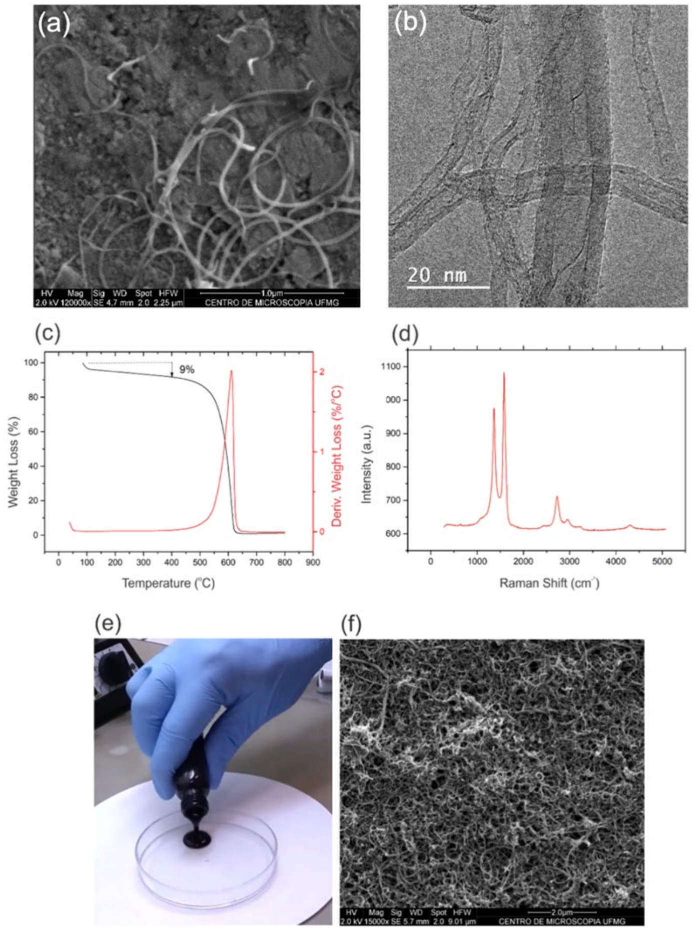

Figure S1: Typical morphology and characteristics of the functionalized multi-walled carbon nanotubes (MWCNTs) used in this work (a) SEM image; (b) TEM image; (c) Thermogravimetric Analyses; (d) Raman spectra; (e) CNT ink; (f) thin film SEM image from spray-deposited ink.

Further characteristics are as follows:

- Viscosity: ca. 1.0 mPa·s
- Tube diameter: 10–30 nm
- Tube length: 1–5 μm
- Sheet resistance (10 μm width): 10^3 Ω/□

Supporting Information – section 2

We performed a geometry optimization varying the length, width, and amount of painted ink layers in the sensor film. Figure S2 depicts the gain response of the sensor as a function of length and width for the case of DI water. The best conditions for our experiments for a 6.5 µL drop volume were 7 mm length and 3 mm width.

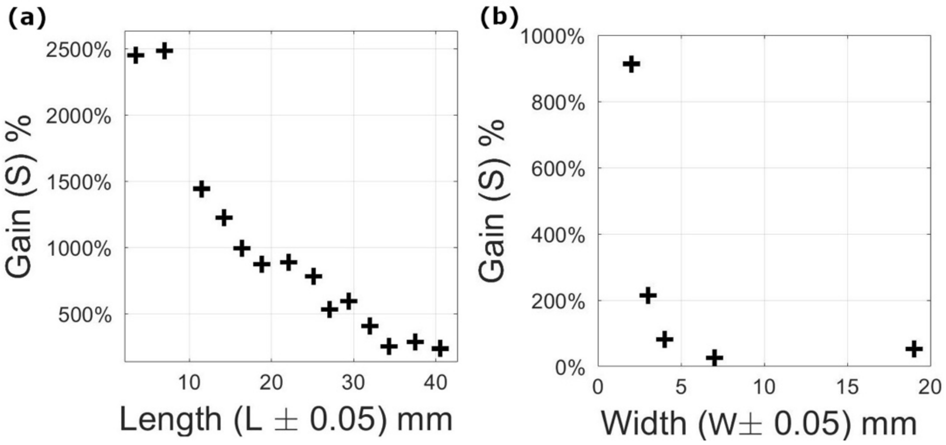

Figure S2: Gain of the sensor film for the case of DI water as a function of the film geometry for a device with 7 mm length and 3 mm width. (a) Length and (b) width. In (a) the width was fixed at 3 mm and in (b) the length was fixed at 10 mm.

To optimize the amount of ink in our devices, we have performed a layer-by-layer painting procedure. Here, the resistance to the painted film is our parameter of optimization. As we increase the amount of ink by increasing the number of painted layers, we are proportionally increasing the conductive paths and decreasing the sensor resistance. We optimized the sensor response by fabricating several sensors with the same width and length, but with an increasing number of layers. Then, we measured the transducer gain as a function of the initial resistance, which is proportional to the number of painted layers. This optimization process can be found in Figure S3. One can observe that as the number of painted layers increases (resistance decreasing), the gain increases. However, experimentally, we noticed that as the number of layers increased, the composite becomes brittle and can crack easily at resistances below 1 kΩ. Therefore, our choice was balanced between the maximum gain and mechanical stability of the sensor (R = 1.26 ± 0.07 kΩ).

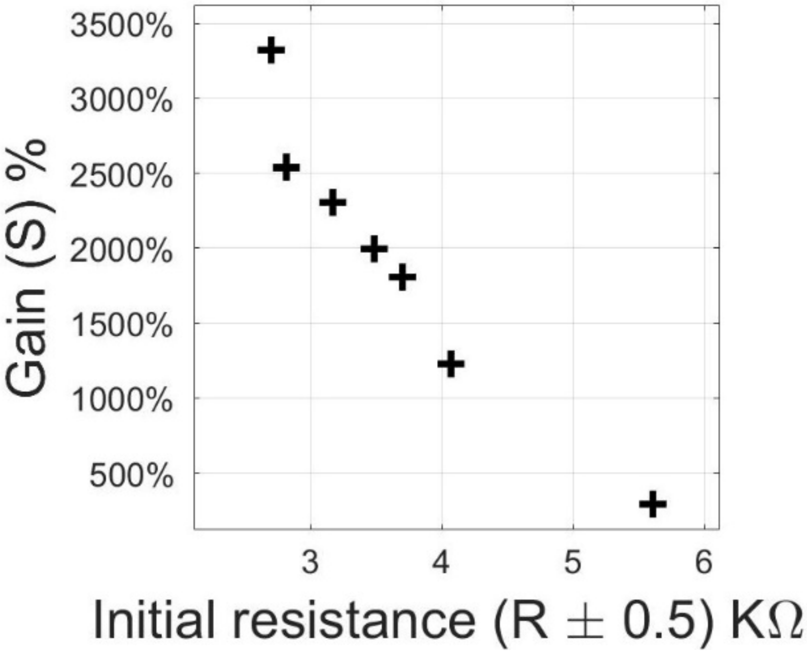

Figure S3: Optimization results for the sensor gain as a function of the initial resistance. The lower the resistance, the higher the number of painted layers.

Supporting Information – section 3

To compare our results with a simple conductive response of the liquid itself, it is necessary to carry out liquid measurements on a substrate with electrical contacts without applying the ink. To achieve this, a device containing two electrodes isolated from each other was fabricated. A voltage of 10 V was applied, and the resistance was monitored in the presence of solvents. The electrical responses of acetone, chloroform, isopropyl alcohol, and ethyl alcohol are shown in Figure S4. The temperature was maintained at 30 °C during the measurements to compare the behavior with that of the MFC/MWCNT sensor.

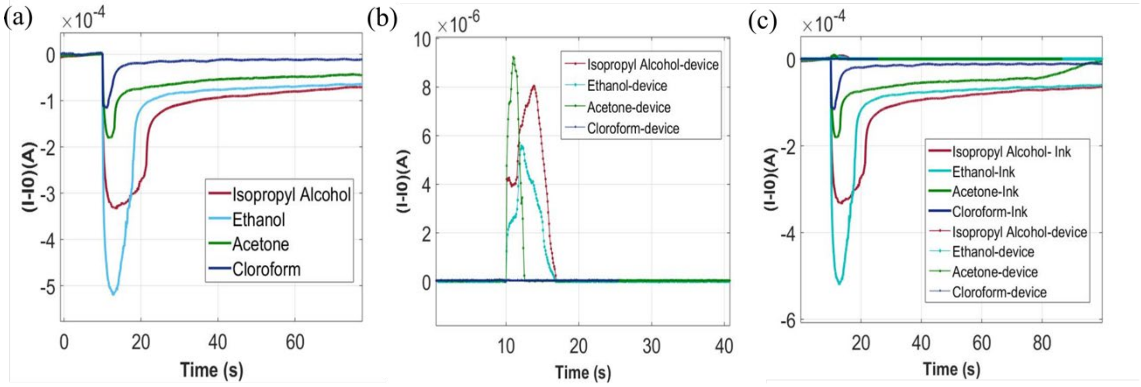

Figure S4: (a) Current variation curves obtained from different organic solvents measured by a transducer based on the MFC/MWCNT ink. (b) Current variation curves for the configuration with electrical contacts without a film. (c) Both curves plotted in the same graph for comparison.

Figure S4a depicts our standard measurement with the ink-printed film, whereas in Figure S4b, we show the electrical response for the system without the sensor film. If one compares Figure S4a and Figure S4b, one can observe that there is a difference of two orders of magnitude in current variation between our sensor in comparison with the blank device. A clearer comparison can be seen in Figure S4c, where both results are placed together in the same graph. Thus, we can conclude that our ink-printed devices distinguish the solvents much better.

Supporting Information – section 4

PCA features variables		
**Variable**	**Definition**	**Algorithm**
Area	area of gain (S) curve data	Area = sum(*S*)
max	maximum value of the gain (S) curve	max = max(*S*)
t_max	time corresponding to the *max* position	tmax =time(max)
slope	slope of a line passing through (0,0) and (tmax, max)	slope = max/tmax
reslope	*slope*^(−1)	reslope = slope − 1
maxdev	maximum value of the numeric differential of gain (S) curve	maxdev = max(diff(*S*))
mindev	minimum value of the numeric differential of gain (S) curve	mindev = min(diff(*S*))
ratio_minmax	ratio of min_dev and max_dev	ratiominmax = maxdev/mindev
WAHM	width of the curve measured at a factor of maximum λ	WAHM = time2(max. * λ) - time1(max. * λ)

References

[1] da Cunha, T. H. R.; de Oliveira, S.; Martins, Icaro, L.; Geraldo, V.; Miquita, D.; Ramos, S., L. M.; Lacerda, R. G.; Ladeira, L. O.; Ferlauto, A. S. *Carbon* **2018,** *133,* 53–61. doi:10.1016/j.carbon.2018.03.014

[2] Castro, V.; Costa, I.; Lopes, M.; Lavall, R.; Figueiredo, K.; Silva, G. *J. Braz. Chem. Soc.* **2017,** *28,* 1158–1166. doi:10.21577/0103-5053.20160274
